# Grazing limits natural biological controls of woody encroachment in Inner Mongolia Steppe

**DOI:** 10.1242/bio.026443

**Published:** 2017-09-13

**Authors:** Hongyu Guo, Linjing Guan, Yinhua Wang, Lina Xie, Chelse M. Prather, Chunguang Liu, Chengcang Ma

**Affiliations:** 1 Tianjin Key Laboratory of Animal and Plant Resistance, College of Life Sciences, Tianjin Normal University, Tianjin 300387, China; 2 College of Life Sciences, Nankai University, Tianjin 300071, China; 3 Department of Biology, University of Dayton, Dayton, OH 45419, USA; 4 College of Environmental Science and Engineering, Nankai University, Tianjin 300350, China

**Keywords:** *Caragana microphylla*, Grazing, Inner Mongolia Steppe, Herbivorous insects, Parasitic plants, Woody encroachment

## Abstract

Woody encroachment in grasslands has become increasingly problematic globally. Grazing by domestic animals can facilitate woody encroachment by reducing competition from herbaceous plants and fire frequency. Herbivorous insects and parasitic plants can each exert forces that result in the natural biological control of encroaching woody plants through reducing seeding of their host woody plants. However, the interplay of grazing and dynamics of herbivorous insects or parasitic plants, and its effects on the potential biological control of woody encroachment in grasslands remains unclear. We investigated the flower and pod damage by herbivorous insects, and the infection rates of a parasitic plant on the shrub *Caragana microphylla*, which is currently encroaching in Inner Mongolia Steppe, under different grazing management treatments (33-year non-grazed, 7-year non-grazed, currently grazed). Our results showed that *Caragana* biomass was highest at the currently grazed site, and lowest at the 33-year non-grazed site. Herbaceous plant biomass followed the opposite pattern, suggesting that grazing is indeed facilitating the encroachment of *Caragana* plants in Inner Mongolia Steppe. Grazing also reduced the abundance of herbivorous insects per *Caragana* flower, numbers of flowers and pods damaged by insect herbivores, and the infection rates of the parasitic plant on *Caragana* plants. Our results suggest that grazing may facilitate woody encroachment in grasslands not only through canonical mechanisms (e.g. competitive release via feeding on grasses, reductions in fires, etc.), but also by limiting natural biological controls of woody plants (herbivorous insects and parasitic plants). Thus, management efforts must focus on preventing overgrazing to better protect grassland ecosystems from woody encroachment.

## Abstract (precis)

**Summary:** Grazing can negatively affect herbivorous insects and parasitic plants on woody plants, and thus limit the effectiveness of their natural biological control effects on woody encroachment.

## INTRODUCTION

The encroachment of woody plants into grasslands has become one of the most significant biological phenomena globally over the last century (Eldridge et al., 2011; Naito and Cairns, 2011). Woody encroachment can have important influences on grassland ecosystem structure and function (Eldridge et al., 2011; Ratajczak et al., 2012). A better understanding of the mechanisms mediating woody encroachment is critical for better prediction of the potential alterations to plant community dynamics, and a better understanding of the consequences of such vegetation compositional shifts in grasslands (Van Auken, 2009).

Researchers have implicated many climatic and ecological factors mediating woody encroachment, such as changes in temperature, precipitation, CO_2_ levels, fire regimes and overgrazing (Archer et al., 1995; Morgan et al., 2007; Coetzee et al., 2008; Gordijn et al., 2013; Kulmatiski and Beard, 2013; Matson and Bart, 2013). The effects of grazing by mammalian animals on woody encroachment across different rangeland ecosystems are complex (Van Auken, 2009; Sankaran et al., 2013). For example, studies in African savannas showed that browsing and trampling by mammalian animals can reduce establishment of woody seedlings (Sankaran et al., 2008, 2013; Staver et al., 2009), while studies in North American semi-arid grasslands showed that high and constant levels of grass herbivory by domestic animals can facilitate woody encroachment (Scholes and Archer, 1997; Van Auken, 2000, 2009). Grazing by domestic animals can reduce biomass of herbaceous plants, resulting in increased resource availability for the establishment of woody plants in grasslands (Coetzee et al., 2008). Grazing by domestic animals also leads to reduction in fire frequency and intensity by reducing the biomass of herbaceous plants (Oba et al., 2000; Roques et al., 2001), favoring woody encroachment in grasslands.

Herbivorous insects and parasitic plants, on the other hand, can exert important control on plant communities, including the spread of species that are woody encroachers. Insect herbivores can strongly affect the establishment and performance of plant species (McPherson, 1993; Prittinen et al., 2003), and thereby have important influence on plant community structure and dynamics (Brown, 1985; Crawley, 1989; Brown and Gange, 1992; Carson and Root, 2000; Borer et al., 2014). Insect herbivores can especially reduce seeding rates of their host plants directly by feeding on, laying on, or developing in seeds, or indirectly by damaging the reproductive parts (e.g. flowers) of the plants, and thereby inhibiting seed formation (Hoffmann and Moran, 1998; Hoffmann et al., 2002; van Klinken et al., 2003; Impson et al., 2011). Through these mechanisms, insect herbivores feeding on flowering plants, including woody plants, can act as natural biological controls on the growth and expansion of these plants. Similar to herbivore effects (Pennings and Callaway, 2002; Pennings and Simpson, 2008), parasitic plants can also have strong impacts on plant community structure and dynamics by limiting the growth and development of their host plants (Pennings and Callaway, 1996; Callaway and Pennings, 1998; Bardgett et al., 2006). Thus, parasitic plants on woody host plants can also function as natural biological controls on woody encroachment. Many previous studies have shown that grazing by large herbivorous animals can affect insect diversity and abundance (Cagnolo et al., 2002; Kruess and Tscharntke, 2002; Hartley et al., 2003; Huntzinger et al., 2008; Zhu et al., 2012). However, the interplay of grazing and dynamics of herbivorous insects or parasitic plants, and its effects on the potential biological control of woody encroachment in grasslands remains unclear.

In this study, we investigated whether natural biological controls of woody encroachment in grasslands, herbivorous insects and parasitic plants on woody plants would be altered by different grazing management approaches. We hypothesized that grazing would negatively impact herbivorous insects and parasitic plants on woody plants, thereby limiting the roles that these natural biological controls play in limiting the expansion of woody plants under grazing, further facilitating woody encroachment in grasslands. To test this hypothesis, we examined how three different grazing management treatments affect the flower and pod damage by herbivorous insects, and the infection rates of a parasitic plant on the encroaching legume shrub, *Caragana microphylla* (hereafter referred to as ‘*Caragana*’), in the semi-arid Inner Mongolia Steppe.

## RESULTS

### Aboveground biomass

**Fig. 1.**
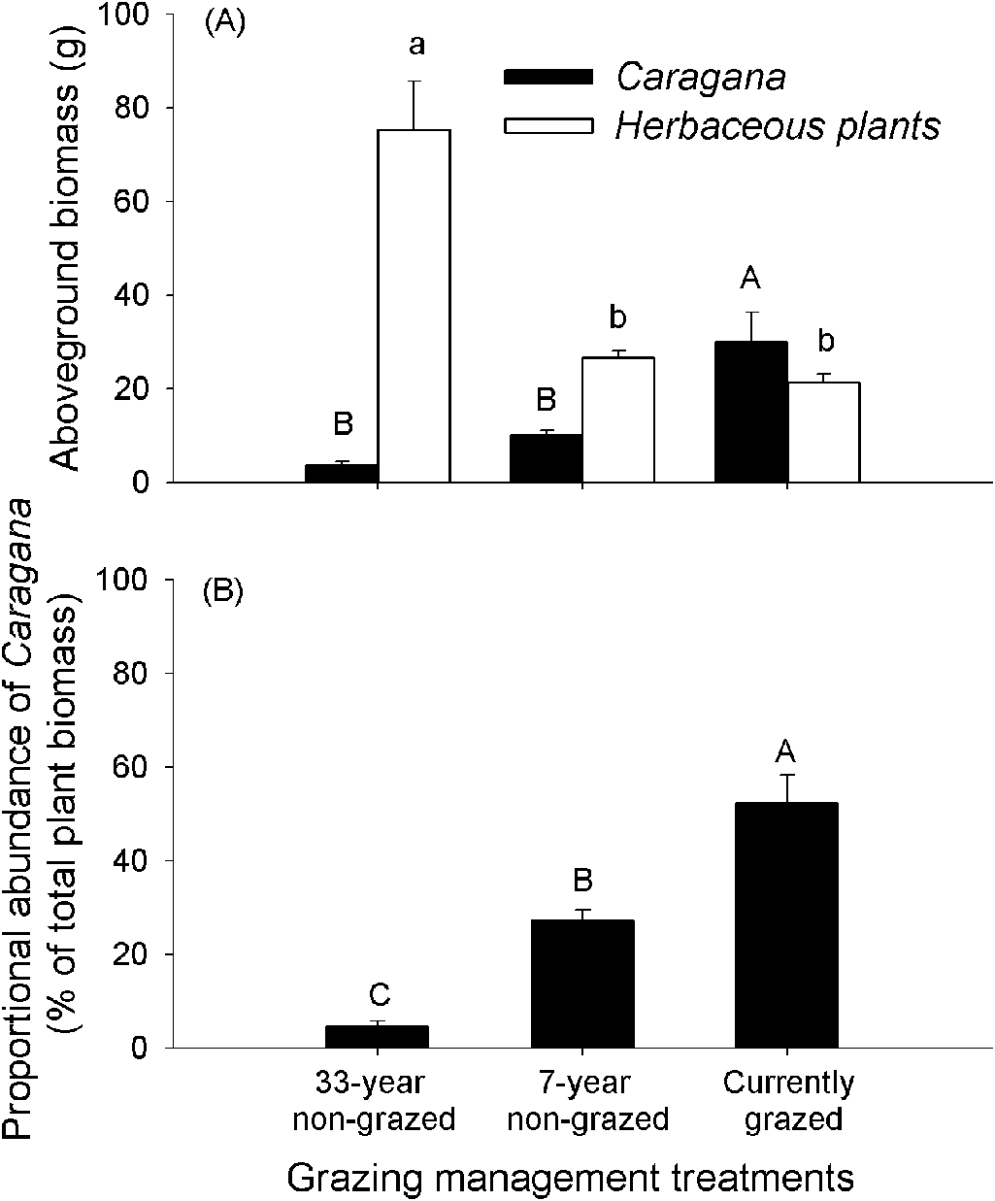
**Aboveground biomass of *Caragana* and herbaceous plants, and proportional abundance of *Caragana*, under different grazing management treatments.** Aboveground biomass of *Caragana* and herbaceous plants (A), and proportional abundance of *Caragana* (% of total plant biomass) (B), in 33-year non-grazed, 7-year non-grazed and currently grazed sites. Data are mean+s.e.m.; *n*=10. Within each panel, shared letters (upper case or lower case) indicate means that are not significantly different from each other (Tukey's HSD tests, significance level of *P*<0.05).

*Caragana* biomass was highest at the currently grazed site, and lowest at the 33-year non-grazed site (*F*_2, 27_=13.10, *P*<0.01) (Fig. 1A). In contrast, the aboveground biomass of herbaceous plants was highest at the 33-year non-grazed site, and lower at the 7-year non-grazed and currently grazed sites (*F*_2, 27_=23.72, *P*<0.01) (Fig. 1A). *Caragana* plants dominated plant communities in terms of biomass at the currently grazed site, but made up a low proportion of the plant community at the 7-year non-grazed and 33-year non-grazed sites (*F*_2, 27_=42.12, *P*<0.01) (Fig. 1B).

### Flower and pod damage by herbivorous insects on *Caragana* plants

**Fig. 2.**
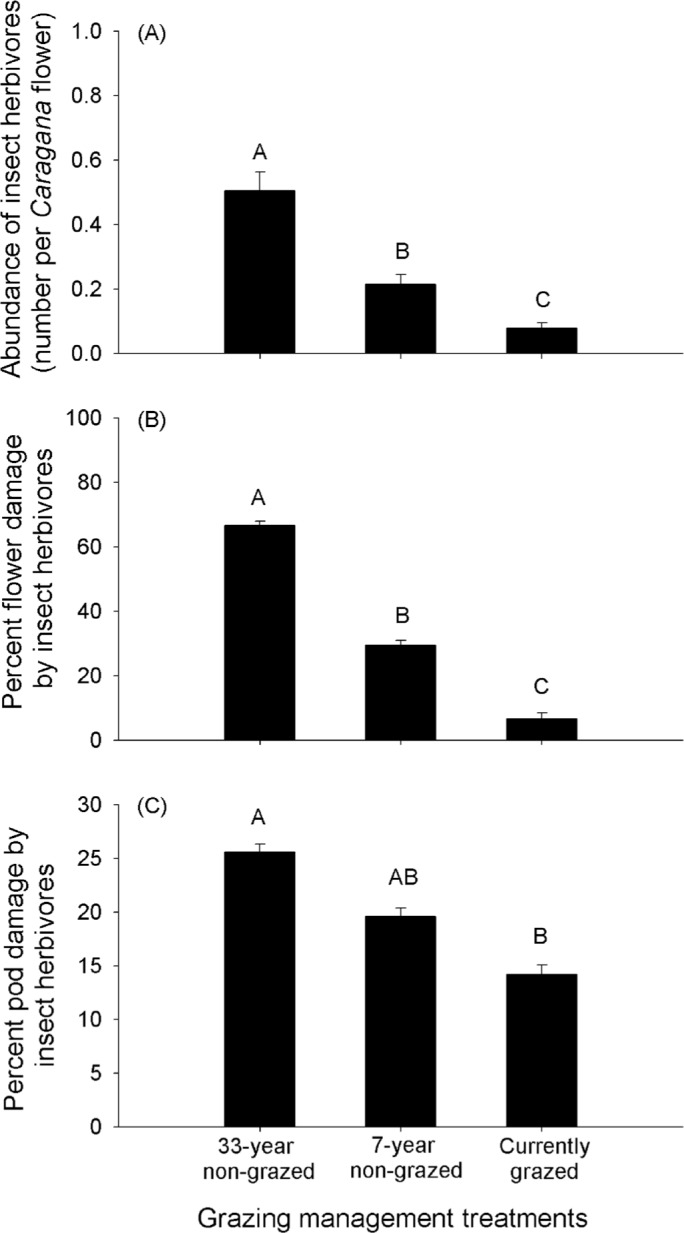
**Abundance of insect herbivores, and percentage of flower and pod damage by insect herbivores, on *Caragana* plants under different grazing management treatments.** Abundance of insect herbivores (number per *Caragana* flower) (A), percent flower damage (B) and percent pod damage (C) by insect herbivores on *Caragana* plants in 33-year non-grazed, 7-year non-grazed and currently grazed sites. Data are mean+s.e.m.; *n*=30 in A, *n*=5 in B, and *n*=5 in C. Within each panel, shared letters indicate means that are not significantly different from each other (Tukey's HSD tests, significance level of *P*<0.05).

Insect abundance was lowest at the currently grazed site and highest at the 33-year non-grazed site (*F*_2, 87_=30.96, *P*<0.01) (Fig. 2A). The percentage of flowers and pods damaged by herbivorous insects on *Caragana* plants followed a similar pattern: the damaged percentage was greatest at the 33-year non-grazed site, intermediate at the 7-year non-grazed site, and lowest at the currently grazed site (flower damage: *F*_2,12_=363.04, *P*<0.01, Fig. 2B; pod damage: *F*_2,12_=49.78, *P*<0.01, Fig. 2C).

### Infection rates of the parasitic plant *Cuscuta* on *Caragana* plants

**Fig. 3.**
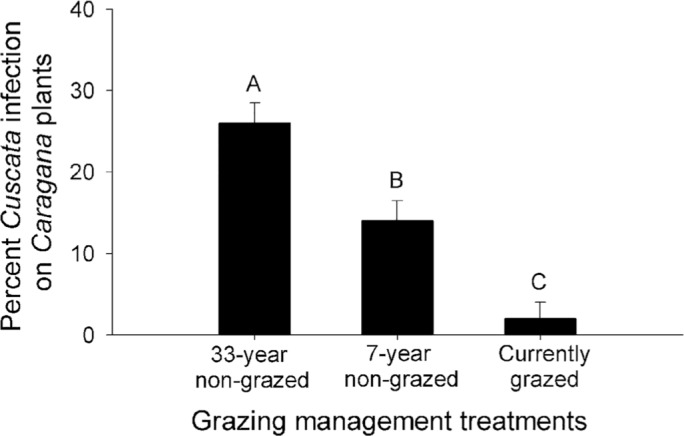
**Infection rates of the parasitic plant *Cuscuta* on *Caragana* plants under different grazing management treatments.** Data are mean+s.e.m.; *n*=5. Within each panel, shared letters indicate means that are not significantly different from each other (Tukey's HSD tests, significance level of *P*<0.05).

The *Caragana* plants had highest levels of *Cuscuta* infection at the 33-year non-grazed site, intermediate infection rates at the 7-year non-grazed site, and lowest infection rates at the currently grazed site (*F*_2,12_=27.00, *P*<0.01) (Fig. 3).

## DISCUSSION

As has been commonly found in studies of woody encroachment and grazing by domestic animals in arid and semi-arid grasslands (Scholes and Archer, 1997; Van Auken, 2000, 2009), we found that continuous stocking of grazing domestic animals enhanced the growth of *Caragana* plants, which are currently encroaching in the semi-arid Inner Mongolia Steppe. The facilitative effects of grazing on *Caragana* encroachment could partially be caused by the commonly recognized mechanisms of reduced competition from herbaceous plants (Coetzee et al., 2008) and reduction in fire frequency under grazing (Oba et al., 2000; Roques et al., 2001). Our results revealed that such facilitation of *Caragana* encroachment in Inner Mongolia Steppe could additionally be caused, in part, by the decreased natural biological controls of herbivorous insects and parasitic plants on *Caragana* encroachment by grazing.

Although grazing by livestock at low intensity and frequency may have little effect on grassland plant communities, high levels of grazing by livestock can lead to reductions in biomass of herbaceous plants and changes in species composition of grasslands, facilitating the transition from grasslands to shrublands or woodlands (Coetzee et al., 2008; Knapp et al., 2008; Good et al., 2013). *Caragana* plants are widely distributed in the semi-arid Inner Mongolia Steppe, and they are relatively resistant to grazing disturbance (Zhang et al., 2006). High levels of grazing by livestock during recent decades in the semi-arid Inner Mongolia Steppe has significantly reduced the cover of herbaceous plants (Li et al., 2013; Peng et al., 2013), which favored the establishment and growth of the relatively grazing-resistant *Caragana* plants, thereby promoting the encroachment of this shrub species in the semi-arid Inner Mongolia Steppe. Additionally, our results showed that the herbivorous insect abundance per flower, and flower and pod damage by herbivorous insects, in *Caragana* plants decreased from the 33-year non-grazed site to the 7-year non-grazed site, and to the currently grazed site, suggesting that grazing had a negative impact on the herbivorous insects on *Caragana* plants, thus limiting natural biological controls of herbivorous insects on the establishment and expansion of *Caragana* plants in Inner Mongolia Steppe.

Many studies have shown that herbivorous insects can reduce seeding of woody host plants by damaging seeds and reproductive parts, or by inhibiting seed formation by debilitating the host plants, which limits the establishment and spread of woody host plants. For example, Hoffmann and Moran (1998) found that two insect species, a bud-feeder, *Trichapion lativentre*, and a seed-feeder, *Rhyssomatus marginatus*, together caused substantial reductions in seeding by the invasive South American tree *Sesbania punicea* in South Africa. Meiners et al. (2000) found that insect herbivory had strong negative effects on the establishment of four tree species in New Jersey, USA. Hoffmann et al. (2002) reported that gall-forming wasp *Trichilogaster* sp. could effectively reduce seed production of the invasive Australian tree *Acacia pycnantha* in South Africa. Chaneton et al. (2010) reported that herbivory by tenebrionid beetles was the main factor limiting the establishment of the woody plant *Austrocedrus chilensis* in a steppe-woodland ecotone in Argentina. Similarly, at the 33-year and 7-year non-grazed sites in our study, the insect herbivores targeting *Caragana* plants reduced the fecundity of *Caragana* plants more strongly by damaging more flowers and pods than at the currently grazed site, indicating relatively strong biological controls of the insect herbivores on the establishment and expansion of *Caragana* plants at the non-grazed sites versus the currently grazed site.

In contrast, the herbivorous insects at the currently grazed site decreased in abundance under the impact of grazing disturbance. Studies in other ecosystems have shown that disturbances can cause local reductions in numbers of insects (Anderson, 1992; Dennis et al., 2008; Schowalter, 2012); and in our case, insects may simply escape from the currently grazed site to avoid the disturbances from grazing by domestic animals and associated anthropogenic activities (Braschler et al., 2009; Schowalter, 2012; Ben-Ari and Inbar, 2013). Thus, with lower abundance of herbivorous insects on *Caragana* plants under grazing disturbance, biological control effects of herbivorous insects on the encroachment of *Caragana* plants decreased at the currently grazed site.

Our results also indicated that grazing also had negative impacts on the parasitic plant *Cuscuta*, thus decreasing the biological control effects of *Cuscuta* on the encroachment of *Caragana* plants in Inner Mongolia Steppe. Previous studies have shown that parasitic plants can have strong effects on the structure and dynamics of plant communities by selectively suppressing the growth of infected plants (Pennings and Callaway, 1996; Callaway and Pennings, 1998; Bardgett et al., 2006). Additionally, parasitic plants often prefer legumes as host plants because of their relatively high nitrogen content (Marvier, 1998; Pennings and Callaway, 2002). At our study sites, the parasitic plant *Cuscuta* mainly attacked *Caragana* plants (leguminous shrub species) and had negative effects on the growth of *Caragana* plants. As the infection rates of *Cuscuta* on *Caragana* plants were higher at the non-grazed sites than those at the currently grazed site, we expect that the growth-suppressing effects of the parasitic plant *Cuscuta* on *Caragana* plants would be stronger at the two non-grazed sites versus the currently grazed site. *Cuscuta* infection might contribute to the lower aboveground biomass of *Caragana* plants at the non-grazed sites versus the currently grazed site. Thus, at the non-grazed sites, the parasitic plant *Cuscuta* could exert a relatively strong biological control on the encroachment of *Caragana* plants, as it suppresses the growth, thereby limiting the reproduction of *Caragana* plants. At the currently grazed site, the infection rate of *Cuscuta* on *Caragana* plants was lower, probably due to grazing by domestic animals on *Cuscuta* plants or seeds (Nicol et al., 2007). This indicated that the parasitic plant *Cuscuta* was negatively affected by grazing disturbance, thereby having limited biological control effects on the encroachment of *Caragana* plants at the currently grazed site.

### Conclusions

Overall, our results demonstrated that grazing can negatively affect the herbivorous insects and the parasitic plant *Cuscuta* on *Caragana* plants, thus limiting the effectiveness of their natural biological control effects on the establishment and expansion of *Caragana* plants in the semi-arid Inner Mongolia Steppe. Grazing would release *Caragana* plants from the pressure of the herbivorous insects and the parasitic plant *Cuscuta* to some extent, which would, in turn, favour the further encroachment of *Caragana* plants in the semi-arid Inner Mongolia Steppe. These results indicated that overgrazing would facilitate woody encroachment at a faster rate than previously expected. Also, the relatively weaker biological control effects of the herbivorous insects and the parasitic plant *Cuscuta* on the expansion of *Caragana* plants at the 7-year non-grazed site versus the 33-year non-grazed site indicated that it might need a long period of time for grassland ecosystems to recover their functioning after overgrazing disturbance. Our results revealed that grazing facilitates woody encroachment in grasslands not only through the canonical mechanisms commonly known, but also by limiting natural biological controls (herbivorous insects and parasitic plants). Thus, management efforts should focus on preventing overgrazing to better protect grassland ecosystems from woody encroachment.

## MATERIALS AND METHODS

### Study sites

Field work was conducted at the Inner Mongolia Grassland Ecosystem Research Station, Chinese Academy of Sciences (IMGERS), which is located in Xilinhaote area on the Inner Mongolia Plateau (43.95°N, 116.07°E). The mean annual temperature and precipitation of Xilinhaote area is ∼2.3°C and ∼286 mm, respectively. The mean annual sunshine time and mean daily solar radiation intensity of Xilinhaote area is ∼2970 h and ∼1.59 kJ cm^−2^ day^−1^, respectively. The vegetation in Xilinhaote area is typical semi-arid Inner Mongolia Steppe, and mainly consists of herbaceous plants, such as the grasses *Leymus chinensis* and *Stipa grandis*. The woody plant, *Caragana microphylla* (referred to as ‘*Caragana*’), is encroaching into these grasslands. *Caragana* plants are legume shrubs, which are relatively resistant to grazing animals (Zhang et al., 2006). In the study area, the herbivorous insects on *Caragana* plants were mainly the beetle species *Mylabris speciosa*, *Mylabris sibirica*, *Epicauta gorhami*, *Epicauta erythrocephala* and *Labidostomis bipunctata*, which mainly prefer to feed on the flowers or pods of *Caragana* plants (H.G. and C.M., unpublished data), and thereby have strong negative effect on seeding of *Caragana* plants. In the study area, the parasitic plant on *Caragana* plants was *Cuscuta chinensis* (referred to as ‘*Cuscuta*’), which generally prefers legume plants as hosts (Marvier, 1998; Pennings and Callaway, 2002; H.G. and C.M., unpublished data), thereby debilitating *Caragana* plants.

In 2012, we conducted field work at three adjacent sites with different grazing management treatments within the IMGERS: the first site (∼3 hectares in size) was a mature grassland community that had been fenced to exclude grazing from large herbivorous animals for 33 years (since 1979), following long-term grazing by local livestock (grazing intensity: ∼two sheep unit ha^−1^; this site is referred to as the ‘33-year non-grazed site’); the second site (∼3 hectares in size) had been fenced to exclude grazing from large animals for 7 years (since 2005), following a long-term grazing by local livestock (grazing intensity: ∼two sheep unit ha^−1^; this site is referred to as the ‘7-year non-grazed site’); and the third site had been grazed by local livestock all the time (grazing intensity: ∼two sheep unit ha^−1^; this site is referred to as the ‘currently grazed site’). The three study sites are geographically close to each other and have relatively uniform environmental conditions and plant species compositions, and experienced similar relatively high level grazing by domestic animals before applying the grazing management treatments (Bai et al., 2010; Xie et al., 2016). No other management measures were applied to these study sites.

### Aboveground biomass

At each study site, we surveyed the aboveground biomass of the herbaceous plants and *Caragana* plants, respectively. In August 2012, we randomly located 10 1×1 m sampling plots within each study site, with a distance of ≥20 m between plots. In each plot, we harvested the aboveground biomass of the herbaceous plants and *Caragana* plants, respectively. Biomass was dried at 65°C to a constant weight and weighed.

### Flower and pod damage by herbivorous insects on *Caragana* plants

In the flowering season, April 2012, we set up five parallel transects (120 m each) with a distance of ≥20 m between transects at each study site, and randomly chose six branches of *Caragana* plants (from six *Caragana* plants) along each transect (in total 30 branches for each site) to count the number of herbivorous insects on each flower in the field, and then calculated the average number of herbivorous insects per flower for each branch (30 averages per study site). We also randomly sampled 100 flowers from *Caragana* plants (∼30 *Caragana* plants) along each of the transects to count the number of flowers with damage by herbivorous insects, and then calculated the percentage of flowers damaged by herbivorous insects for each transect (five replicates per study site). We visited the study sites again in August 2012, when the pods of *Caragana* plants had matured. At each study site, we set up five parallel transects (120 m each) with a distance of ≥20 m between transects, and randomly sampled 100 pods from *Caragana* plants (∼30 *Caragana* plants) along each transect to count the number of pods with damage by herbivorous insects, and then calculated the percentage of pods damaged by herbivorous insects for each transect (five replicates per study site).

### Infection rates of the parasitic plant *Cuscuta* on *Caragana* plants

At each study site, we studied the infection rates of the parasitic plant *Cuscuta* on *Caragana* plants. In August 2012, we set up five parallel transects (100 m each) within each study site, with a distance of ≥20 m between transects. Along each transect, we sampled *Caragana* plants approximately every 10 m (10 *Caragana* plants sampled per transect), and counted the number of *Caragana* plants infected by *Cuscuta*, and then calculated the infection rate of *Cuscuta* plants on *Caragana* plants for each transect (five replicates per study site).

### Data analysis

Data analyses were performed with JMP9 statistical software (SAS Institute, 2010). We used ANOVAs to test the differences of means among the grazing management treatments for all variables (all data met the assumptions of normality and homogeneity of variances), and then performed post-hoc Tukey's HSD tests to explicitly compare the means between the grazing management treatments for all the variables.

## References

[BIO026443C1] Anderson N. H. (1992). Influence of disturbance on insect communities in Pacific Northwest streams. *Hydrobiologia* 248, 79-92. 10.1007/BF00008887

[BIO026443C2] Archer S., Schimel D. S. and Holland E. A. (1995). Mechanisms of shrubland expansion: land use, climate or CO_2_? *Clim. Change* 29, 91-99. 10.1007/BF01091640

[BIO026443C3] Bai Y., Wu J., Clark C. M., Naeem S., Pan Q., Huang J., Zhang L. and Han X. (2010). Tradeoffs and thresholds in the effects of nitrogen addition on biodiversity and ecosystem functioning: evidence from inner Mongolia Grasslands. *Glob. Change Biol.* 16, 358-372. 10.1111/j.1365-2486.2009.01950.x

[BIO026443C4] Bardgett R. D., Smith R. S., Shiel R. S., Peacock S., Simkin J. M., Quirk H. and Hobbs P. J. (2006). Parasitic plants indirectly regulate below-ground properties in grassland ecosystems. *Nature* 439, 969-972. 10.1038/nature04197 16495998

[BIO026443C5] Ben-Ari M. and Inbar M. (2013). When herbivores eat predators: predatory insects effectively avoid incidental ingestion by mammalian herbivores. *PLoS ONE* 8, e56748 10.1371/journal.pone.0056748 23424674 PMC3570466

[BIO026443C6] Borer E. T., Seabloom E. W., Gruner D. S., Harpole W. S., Hillebrand H., Lind E. M., Adler P. B., Alberti J., Anderson T. M., Bakker J. D. et al. (2014). Herbivores and nutrients control grassland plant diversity via light limitation. *Nature* 508, 517-520. 10.1038/nature13144 24670649

[BIO026443C7] Braschler B., Marini L., Thommen G. H. and Baur B. (2009). Effects of small-scale grassland fragmentation and frequent mowing on population density and species diversity of orthopterans: a long-term study. *Ecol. Entomol.* 34, 321-329. 10.1111/j.1365-2311.2008.01080.x

[BIO026443C8] Brown V. K. (1985). Insect herbivores and plant succession. *Oikos* 44, 17-22. 10.2307/3544037

[BIO026443C9] Brown V. K. and Gange A. C. (1992). Secondary plant succession: how is it modified by insect herbivory? *Vegetatio* 101, 3-13. 10.1007/BF00031910

[BIO026443C10] Cagnolo L., Molina S. I. and Valladares G. R. (2002). Diversity and guild structure of insect assemblages under grazing and exclusion regimes in a montane grassland from Central Argentina. *Biodivers. Conserv.* 11, 407-420. 10.1023/A:1014861906082

[BIO026443C11] Callaway R. M. and Pennings S. C. (1998). Impact of a parasitic plant on the zonation of two salt marsh perennials. *Oecologia* 114, 100-105. 10.1007/s004420050425 28307547

[BIO026443C12] Carson W. P. and Root R. B. (2000). Herbivory and plant species coexistence: community regulation by an outbreaking phytophagous Insect. *Ecol. Monogr.* 70, 73-99. 10.1890/0012-9615(2000)070[0073:HAPSCC]2.0.CO;2

[BIO026443C13] Chaneton E. J., Noemi Mazía C. and Kitzberger T. (2010). Facilitation vs. apparent competition: insect herbivory alters tree seedling recruitment under nurse shrubs in a steppe–woodland ecotone. *J. Ecol.* 98, 488-497. 10.1111/j.1365-2745.2009.01631.x

[BIO026443C14] Coetzee B. W. T., Tincani L., Wodu Z. and Mwasi S. M. (2008). Overgrazing and bush encroachment by *Tarchonanthus camphoratus* in a semi-arid savanna. *Afr. J. Ecol.* 46, 449-451. 10.1111/j.1365-2028.2007.00842.x

[BIO026443C15] Crawley M. J. (1989). Insect herbivores and plant population dynamics. *Annu. Rev. Entomol.* 34, 531-562. 10.1146/annurev.en.34.010189.002531

[BIO026443C16] Dennis P., Skartveit J., McCracken D. I., Pakeman R. J., Beaton K., Kunaver A. and Evans D. M. (2008). The effects of livestock grazing on foliar arthropods associated with bird diet in upland grasslands of Scotland. *J. Appl. Ecol.* 45, 279-287. 10.1111/j.1365-2664.2007.01378.x

[BIO026443C17] Eldridge D. J., Bowker M. A., Maestre F. T., Roger E., Reynolds J. F. and Whitford W. G. (2011). Impacts of shrub encroachment on ecosystem structure and functioning: towards a global synthesis. *Ecol. Lett.* 14, 709-722. 10.1111/j.1461-0248.2011.01630.x 21592276 PMC3563963

[BIO026443C18] Good M. K., Schultz N. L., Tighe M., Reid N. and Briggs S. V. (2013). Herbaceous vegetation response to grazing exclusion in patches and inter-patches in semi-arid pasture and woody encroachment. *Agric. Ecosyst. Environ.* 179, 125-132. 10.1016/j.agee.2013.08.002

[BIO026443C19] Gordijn P. J., Rice E. and Ward D. (2013). The effects of fire on woody plant encroachment are exacerbated by succession of trees of decreased palatability. *S. Afr. J. Bot.* 86, 142-142 10.1016/j.sajb.2013.02.018

[BIO026443C20] Hartley S. E., Gardner S. M. and Mitchell R. J. (2003). Indirect effects of grazing and nutrient addition on the hemipteran community of heather moorlands. *J. Appl. Ecol.* 40, 793-803. 10.1046/j.1365-2664.2003.00846.x

[BIO026443C21] Hoffmann J. H. and Moran V. C. (1998). The population dynamics of an introduced tree, *Sesbania punicea*, in South Africa, in response to long-term damage caused by different combinations of three species of biological control agents. *Oecologia* 114, 343-348. 10.1007/s004420050456 28307777

[BIO026443C22] Hoffmann J. H., Impson F. A. C., Moran V. C. and Donnelly D. (2002). Biological control of invasive golden wattle trees (*Acacia pycnantha*) by a gall wasp, *Trichilogaster* sp. (*Hymenoptera*: *Pteromalidae*), in South Africa. *Biol. Control* 25, 64-73. 10.1016/S1049-9644(02)00039-7

[BIO026443C23] Huntzinger M., Karban R. and Cushman J. H. (2008). Negative effects of vertebrate herbivores on invertebrates in a coastal dune community. *Ecology* 89, 1972-1980. 10.1890/07-0834.1 18705383

[BIO026443C24] Impson F. A. C., Kleinjan C. A., Hoffmann J. H., Post J. A. and Wood A. R. (2011). Biological control of Australian *Acacia* species and *Paraserianthes lophantha* (Willd.) Nielsen (*Mimosaceae*) in South Africa. *Afr. Entomol.* 19, 186-207. 10.4001/003.019.0210

[BIO026443C25] Knapp A. K., Briggs J. M., Collins S. L., Archer S. R., Bret-Harte M. S., Ewers B. E., Peters D. P., Young D. R., Shaver G. R., Pendall E. et al. (2008). Shrub encroachment in North American grasslands: shifts in growth form dominance rapidly alters control of ecosystem carbon inputs. *Glob. Change Biol.* 14, 615-623. 10.1111/j.1365-2486.2007.01512.x

[BIO026443C26] Kruess A. and Tscharntke T. (2002). Contrasting responses of plant and insect diversity to variation in grazing intensity. *Biol. Conserv.* 106, 293-302. 10.1016/S0006-3207(01)00255-5

[BIO026443C27] Kulmatiski A. and Beard K. H. (2013). Woody plant encroachment facilitated by increased precipitation intensity. *Nat. Clim. Change* 3, 833-837. 10.1038/nclimate1904

[BIO026443C28] Li X. Y., Zhang S. Y., Peng H. Y., Hu X. and Ma Y. J. (2013). Soil water and temperature dynamics in shrub-encroached grasslands and climatic implications: Results from Inner Mongolia steppe ecosystem of north China. *Agric. For. Meteorol.* 171, 20-30. 10.1016/j.agrformet.2012.11.001

[BIO026443C29] Marvier M. (1998). A mixed diet improves performance and herbivore resistance of a parasitic plant. *Ecology* 79, 1272-1280. 10.1890/0012-9658(1998)079[1272:AMDIPA]2.0.CO;2

[BIO026443C30] Matson E. and Bart D. (2013). Interactions among fire legacies, grazing and topography predict shrub encroachment in post-agricultural páramo. *Landsc. Ecol.* 28, 1829-1840. 10.1007/s10980-013-9926-5

[BIO026443C31] McPherson G. R. (1993). Effects of herbivory and herb interference on oak establishment in a semi-arid temperate savanna. *J. Veg. Sci.* 4, 687-692. 10.2307/3236134

[BIO026443C32] Meiners S. J., Handel S. N. and Pickett S. T. A. (2000). Tree seedling establishment under insect herbivory: edge effects and inter- annual variation. *Plant Ecol.* 151, 161-170. 10.1023/A:1026509529570

[BIO026443C33] Morgan J. A., Milchunas D. G., LeCain D. R., West M. and Mosier A. R. (2007). Carbon dioxide enrichment alters plant community structure and accelerates shrub growth in the shortgrass steppe. *Proc. Natl Acad. Sci. USA* 104, 14724-14729. 10.1073/pnas.0703427104 17785422 PMC1964545

[BIO026443C34] Naito A. T. and Cairns D. M. (2011). Patterns and processes of global shrub expansion. *Prog. Phys. Geography* 35, 423-442. 10.1177/0309133311403538

[BIO026443C35] Nicol J., Muston S., D'Santos P., McCarthy B. and Zukowski S. (2007). Impact of sheep grazing on the soil seed bank of a managed ephemeral wetland: implications for management. *Aust. J. Bot.* 55, 103-109. 10.1071/BT04137

[BIO026443C36] Oba G., Post E., Syvertsen P. O. and Stenseth N. C. (2000). Bush cover and range condition assessments in relation to landscape and grazing in southern Ethiopia. *Landsc. Ecol.* 15, 535-546. 10.1023/A:1008106625096

[BIO026443C37] Peng H.-Y., Li X.-Y., Li G.-Y., Zhang Z.-H., Zhang S.-Y., Li L., Zhao G.-Q., Jiang Z.-Y. and Ma Y.-J. (2013). Shrub encroachment with increasing anthropogenic disturbance in the semiarid Inner Mongolian grasslands of China. *CATENA* 109, 39-48. 10.1016/j.catena.2013.05.008

[BIO026443C38] Pennings S. C. and Callaway R. M. (1996). Impact of a parasitic plant on the structure and dynamics of salt marsh vegetation. *Ecology* 77, 1410-1419. 10.2307/2265538

[BIO026443C39] Pennings S. C. and Callaway R. M. (2002). Parasitic plants: parallels and contrasts with herbivores. *Oecologia* 131, 479-489. 10.1007/s00442-002-0923-7 28547541

[BIO026443C40] Pennings S. C. and Simpson J. C. (2008). Like herbivores, parasitic plants are limited by host nitrogen content. *Plant Ecol.* 196, 245-250. 10.1007/s11258-007-9348-z

[BIO026443C41] Prittinen K., Pusenius J., Koivunoro K., Rousi M. and Roininen H. (2003). Mortality in seedling populations of Silver Birch: genotypic variation and herbivore effects. *Funct. Ecol.* 17, 658-663. 10.1046/j.1365-2435.2003.00777.x

[BIO026443C42] Ratajczak Z., Nippert J. B. and Collins S. L. (2012). Woody encroachment decreases diversity across North American grasslands and savannas. *Ecology* 93, 697-703. 10.1890/11-1199.1 22690619

[BIO026443C43] Roques K. G., O'Connor T. G. and Watkinson A. R. (2001). Dynamics of shrub encroachment in an African savanna: relative influences of fire, herbivory, rainfall and density dependence. *J. Appl. Ecol.* 38, 268-280. 10.1046/j.1365-2664.2001.00567.x

[BIO026443C44] Sankaran M., Ratnam J. and Hanan N. (2008). Woody cover in African savannas: the role of resources, fire and herbivory. *Glob. Ecol. Biogeogr.* 17, 236-245. 10.1111/j.1466-8238.2007.00360.x

[BIO026443C45] Sankaran M., Augustine D. J. and Ratnam J. (2013). Native ungulates of diverse body sizes collectively regulate long-term woody plant demography and structure of a semi-arid savanna. *J. Ecol.* 101, 1389-1399. 10.1111/1365-2745.12147

[BIO026443C46] SAS Institute. (2010). *JMP Statistical Software Package. Version 9*. Cary, North Carolina, USA: SAS Institute.

[BIO026443C47] Scholes R. J. and Archer S. R. (1997). Tree-grass interactions in savannas. *Annu. Rev. Ecol. Syst.* 28, 517-544. 10.1146/annurev.ecolsys.28.1.517

[BIO026443C48] Schowalter T. D. (2012). Insect responses to major landscape-level disturbance. *Annu. Rev. Entomol.* 57, 1-20. 10.1146/annurev-ento-120710-100610 21888518

[BIO026443C49] Staver A. C., Bond W. J., Stock W. D., van Rensburg S. J. and Waldram M. S. (2009). Browsing and fire interact to suppress tree density in an African savanna. *Ecol. Appl.* 19, 1909-1919. 10.1890/08-1907.1 19831079

[BIO026443C50] Van Auken O. W. (2000). Shrub invasions of North American semiarid grasslands. *Annu. Rev. Ecol. Syst.* 31, 197-215. 10.1146/annurev.ecolsys.31.1.197

[BIO026443C51] Van Auken O. W. (2009). Causes and consequences of woody plant encroachment into western North American grasslands. *J. Environ. Manag.* 90, 2931-2942. 10.1016/j.jenvman.2009.04.023 19501450

[BIO026443C52] van Klinken R. D., Fichera G. and Cordo H. (2003). Targeting biological control across diverse landscapes: the release, establishment, and early success of two insects on mesquite (*Prosopis* spp.) insects in Australian rangelands. *Biol. Control* 26, 8-20. 10.1016/S1049-9644(02)00107-X

[BIO026443C53] Xie L., Chen W., Gabler C. A., Han L., Guo H., Chen Q., Ma C. and Gu S. (2016). Effects of grazing intensity on seed production of *Caragana stenophylla* along a climatic aridity gradient in the Inner Mongolia Steppe, China. *J. Arid Land* 8, 890-898. 10.1007/s40333-016-0050-7

[BIO026443C54] Zhang Z., Wang S.-P., Nyren P. and Jiang G.-M. (2006). Morphological and reproductive response of *Caragana microphylla* to different stocking rates. *J. Arid Environ.* 67, 671-677. 10.1016/j.jaridenv.2006.03.015

[BIO026443C55] Zhu H., Wang D., Wang L., Bai Y., Fang J. and Liu J. (2012). The effects of large herbivore grazing on meadow steppe plant and insect diversity. *J. Appl. Ecol.* 49, 1075-1083. 10.1111/j.1365-2664.2012.02195.x

